# Zebrafish 20β-Hydroxysteroid Dehydrogenase Type 2 Is Important for Glucocorticoid Catabolism in Stress Response

**DOI:** 10.1371/journal.pone.0054851

**Published:** 2013-01-22

**Authors:** Janina Tokarz, William Norton, Gabriele Möller, Martin Hrabé de Angelis, Jerzy Adamski

**Affiliations:** 1 Helmholtz Zentrum München, German Research Center for Environmental Health, Institute of Experimental Genetics, Genome Analysis Center, Neuherberg, Germany; 2 Centre Nationale de la Recherche Scientifique, Zebrafish Neurogenetics, Gif sur Yvette, France; 3 Lehrstuhl für Experimentelle Genetik, Technische Universität München, Freising-Weihenstephan, Germany; National Cancer Institute, United States of America

## Abstract

Stress, the physiological reaction to a stressor, is initiated in teleost fish by hormone cascades along the hypothalamus-pituitary-interrenal (HPI) axis. Cortisol is the major stress hormone and contributes to the appropriate stress response by regulating gene expression after binding to the glucocorticoid receptor. Cortisol is inactivated when 11β-hydroxysteroid dehydrogenase (HSD) type 2 catalyzes its oxidation to cortisone. In zebrafish, *Danio rerio*, cortisone can be further reduced to 20β-hydroxycortisone. This reaction is catalyzed by 20β-HSD type 2, recently discovered by us. Here, we substantiate the hypothesis that 20β-HSD type 2 is involved in cortisol catabolism and stress response. We found that *hsd11b2* and *hsd20b2* transcripts were up-regulated upon cortisol treatment. Moreover, a cortisol-independent, short-term physical stressor led to the up-regulation of *hsd11b2* and *hsd20b2* along with several HPI axis genes. The morpholino-induced knock down of *hsd20b2* in zebrafish embryos revealed no developmental phenotype under normal culture conditions, but prominent effects were observed after a cortisol challenge. Reporter gene experiments demonstrated that 20β-hydroxycortisone was not a physiological ligand for the zebrafish glucocorticoid or mineralocorticoid receptor but was excreted into the fish holding water. Our experiments show that 20β-HSD type 2, together with 11β-HSD type 2, represents a short pathway in zebrafish to rapidly inactivate and excrete cortisol. Therefore, 20β-HSD type 2 is an important enzyme in stress response.

## Introduction

Stress is an ancient evolutionary system that enables fast reflexive actions to cope with environmental stressors or dangerous situations, e.g., predators. In a life-threatening situation, an organism must be ready to act. Instinctive behaviors (fight-or-flight) are preferred over a precise but tedious evaluation of the situation. In vertebrates, stressors are perceived in the hypothalamus via input from the central and peripheral nervous system and are then transmitted by hormone cascades of the hypothalamus-pituitary-adrenal axis (HPA axis) to the body for instant activation [1], [2]. Because teleost fish do not possess a discrete adrenal gland but do possess the homologous interrenal cells embedded in the head kidney, signal transduction occurs via the hypothalamus-pituitary-interrenal axis (HPI axis). The first hormone secreted by the hypothalamus is corticotropin releasing hormone (CRH), which stimulates the corticotropic and melanotropic cells of the pituitary [3] to cleave the large proopiomelanocortin protein (POMC) [4]. This cleavage allows adrenocorticotropic hormone (ACTH) to be secreted into the circulation and to bind the melanocortin 2 receptor (MC2R) on the surface of the interrenal cells in teleost fish [5]. Upon this stimulus, the interrenal cells enclosed in the head kidney synthesize and release cortisol into the circulation [6]. The *de novo* biosynthesis of cortisol in fish begins with the transport of cholesterol into the mitochondria, which is mediated by steroidogenic acute regulatory protein (StAR). Next, side chain cleavage of cholesterol is catalyzed by monooxygenase P450scc (Cyp11a1). Afterwards, 17alpha-hydroxylation (Cyp17), 3β-hydroxysteroid dehydrogenation (Hsd3b), and 21-hydroxylation (Cyp21a1) occur prior to the last step in cortisol biosynthesis, which is 11-hydroxylation mediated by 11-hydroxylase (Cyp11c1) [7].

As the major stress hormone, cortisol contributes to a general activation of the organism and the preparation of the appropriate stress response. The stimulation of gluconeogenesis in the liver [7], [8], proteolytic processes in the muscle, and lipolysis in the adipose tissues [7] increase the plasma glucose concentration to provide energy for stress responses. Furthermore, cortisol controls ionic and osmotic regulation, e.g., in adaptation of euryhaline teleost fish to sea water [9], and regulates immune functions, growth, and behavior [7], [10]. Although the functions of cortisol during embryonic development have not yet been entirely unraveled, the zygote is maternally supplied with the hormone in several teleost species (see [11] and references therein), indicating its importance during embryonic development [1], [12]. Indeed, recent studies focusing on zebrafish demonstrate a crucial role for cortisol in development [13]–[15] and ion transport [16].

Because it is a potent hormone that regulates a variety of vital functions, chronic exposure to elevated cortisol concentrations during either embryonic development or adult life causes several adverse effects. Teleost fish embryos exposed to excess cortisol show increased mortality and developmental defects [12], [17], [18]. Chronic stress and elevated cortisol concentrations impact growth, reproduction, immune functions, and increased mortality caused by diseases in adult fish [19]–[22].

Cortisol mediates its effects by binding to members of the nuclear receptor family, which then translocate to the nucleus and regulate the transcription of target genes. In humans and teleost fish, cortisol is the unique and highly affine ligand of the glucocorticoid receptor α (GRα) [7], [23], [24]. Additionally, in humans the affinity of cortisol for the mineralocorticoid receptor (MR) is comparable to that of aldosterone, the native MR ligand [24]. Because cortisol is more abundant in the circulation than aldosterone, permanent MR activation by cortisol in mineralocorticoid-sensitive tissues can be avoided by the expression of the cortisol-inactivating enzyme 11β-hydroxysteroid dehydrogenase (HSD) type 2 [25], [26]. In teleost fish, the native ligand of MR is not yet known because fish lack an aldosterone biosynthetic pathway [27]. However, recent studies provide evidence that 11-deoxycorticosterone might be the ligand for the teleostean MR [28], [29].

Cortisol levels are controlled by the ratio of *de novo* synthesis to catabolism by the action of the respective enzymes involved. The first enzyme in cortisol inactivation and catabolism is 11β-HSD type 2, which catalyzes the conversion of cortisol to cortisone [30]–[32]. Downstream enzymes in the catabolic pathway include reductases, oxidoreductases, and hydroxylases that yield a broad spectrum of glucocorticoids [7]. Analyses of the glucocorticoids excreted from fish identified tetrahydro-derivatives of cortisol and cortisone, including 20β-cortolone, 5β-dihydroxycortisone, cortisol, and cortisone [33]–[35]. Another putatively excreted glucocorticoid, 4-pregnen-17α,20β,21-triol-3,11-dione (20β-hydroxycortisone), was recently identified by us as the product of a cortisone reduction catalyzed by the novel enzyme 20β-HSD type 2 in various fish species [36]. Following the analyses of its expression in zebrafish and the evaluation of its kinetic parameters, we hypothesized a role for 20β-HSD type 2 in cortisol catabolism and stress response in concert with 11β-HSD type 2 [36]. To confirm this hypothesis, we investigated the regulation of both 11β-HSD type 2 and 20β-HSD type 2 in zebrafish embryos upon cortisol treatment and after physical stress. We also elucidated the physiological implications of a morpholino-induced knock down of 20β-HSD type 2. Furthermore, we determined the ability of 20β-hydroxycortisone to activate the GRα and MR, and analyzed conjugated and free glucocorticoids released into the fish holding water.

## Materials and Methods

### Fish Stocks

All experiments were performed on embryos of the zebrafish AB-EK strain and were in accordance with the EU directive 2010/63/EU as well as the German Animal Welfare Act. Standard fish-keeping protocols were followed [37], and embryos were obtained by natural spawning. Embryos were staged according to Kimmel *et* *al.* [38].

### Injection of Morpholino Antisense Nucleotides into Zebrafish Embryos

**Figure 1:**
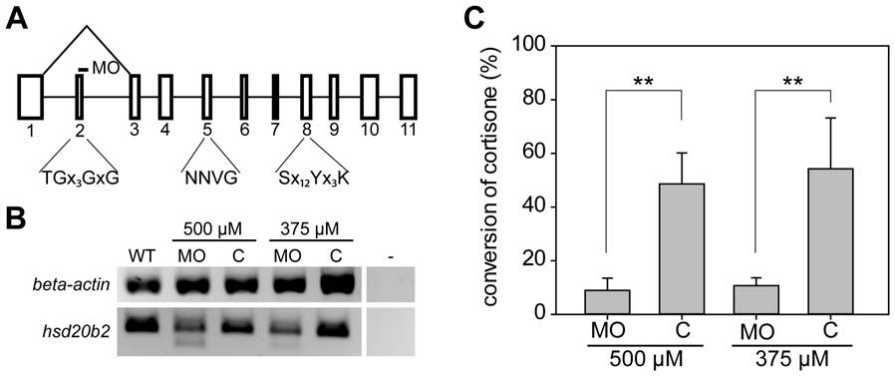
Analyses of knock down efficiency of an *hsd20b2* splicing morpholino reveal reduction of enzymatic activity. (A) In the genomic structure of zebrafish *hsd20b2*, exons are indicated by boxes and numbered. Below, the important short-chain dehydrogenase/reductase motifs (single letter amino acid code) are denoted. ‘x’ denotes any amino acid residue, and when present, the subsequent number indicates the number of×residues. The splice site targeted by the morpholino is indicated by a dash. Morpholino-induced mis-splicing is illustrated by the triangle above the genomic structure. Exons are to scale, whereas the space between the exons does not reflect the respective intron size. (B) The analyses of morpholino efficiency at the mRNA level by RT-PCR using primers that prime within the first and fourth exons demonstrate mis-splicing in morpholino-injected fish (MO). The smaller PCR product is absent from samples of the control morpholino-injected fish embryos (C). The respective morpholino concentration used is denoted. β-actin controls were included for normalization. (C) The knock down efficiency was analyzed by assaying the enzymatic activity that converts cortisone to 20β-hydroxycortisone in morpholino (MO)- and control morpholino (C)-injected fish. The respective morpholino concentration used is denoted and mean values with standard deviations from four biological replicates are presented. Significant levels are indicated: ** p<0.01.

Morpholino antisense nucleotides (Gene Tools, Philomath, Oregon, USA) were designed to target the donor splice site of exon 2 of *hsd20b2* mRNA (Figure 1 A). The splicing morpholino had the following sequence (5′-GAATAAAATACTGACCTCTTCAGCA-3′), while the control morpholino contained five mismatches (5′-GAATAAAATAgTcAgCTCTTgAcCA-3′). The morpholinos were dissolved in water to final concentrations of 375 µM and 500 µM. Embryos at the 1-cell stage were injected with approximately 4 nL of morpholino into the yolk using a FemtoJet injector equipped with a micromanipulator (Eppendorf, Hamburg, Germany). The embryos were kept in egg water (60 µg/L sea salt; Instant Ocean, Miami, Florida, USA) at 28.5°C and were frequently monitored for viability and developmental phenotypes. At 48 hpf, viable embryos were collected and snap-frozen in liquid nitrogen for RNA isolation and enzymatic assays.

### Challenging Zebrafish Embryos with Cortisol

Zebrafish embryos (wild-type and morpholino-injected) were kept in egg water at 28.5°C and treated with cortisol in two different setups. For the 3–72 hpf exposure, zebrafish embryos were challenged with cortisol (Sigma-Aldrich, Hamburg, Germany; final concentrations of 10, 25, 50, 75, and 100 mg/L (equivalent to 27.5, 69.0, 137.9, 206.9, and 275.9 µM, respectively), which was dissolved in dimethylformamide (DMF; Merck, Darmstadt, Germany) prior to its addition to the egg water. The amount of DMF was used in a range of 0.1–1%. Treatment began at 3 hpf and continued until 72 hpf. Samples for RNA isolation contained 30 embryos each and were collected at 24 hpf, 48 hpf, and 72 hpf and snap-frozen in liquid nitrogen. At each sampling point, water was exchanged with fresh egg water containing the respective treatment regimen. Controls, including a “no treatment” control of egg water alone and solvent controls with the appropriate amount of DMF were run concurrently with each experiment. For the 3–24 hpf exposure, the egg water was exchanged at 3 hpf with egg water containing 50 mg/L (137.9 µM) cortisol. At 24 hpf, samples were collected and the water was exchanged with normal egg water. Controls, including a “no treatment” control of egg water alone and a “solvent control” of 0.1% DMF until 24 hpf, were run concurrently with each experiment. The embryos were monitored throughout the experiments for viability and developmental abnormalities. In both experiments, three samples were collected per sampling point.

### Challenging Zebrafish Larvae with a Physical Stressor

Larvae used for the analyses of stress responses were 5 dpf because the stress axis is fully functional by this age [11]. Six pools of 50 larvae each were subjected to a stressor regimen consisting of swirling the fish for 30 sec in a 25 mL glass beaker containing 5 mL of egg water at 600 rpm. Subsequently, the larvae were placed in a 28.5°C incubator and sampled at different time points (5 min, 10 min, 20 min, 30 min, and 60 min) after the stress. Control samples were taken before applying the stressor. Pools of 50 fish were divided into two aliquots and snap-frozen in liquid nitrogen. One aliquot of each pool was used for RNA isolation.

### Preparation of Embryos for Phenotypic Evaluation

Zebrafish embryos (wild-type and morpholino-injected) at 48 hpf and 72 hpf were fixed overnight in 4% paraformaldehyde (Sigma-Aldrich, Hamburg, Germany) in PBS at 4°C, washed twice in PBT (0.1% Tween-20 (Sigma-Aldrich, Hamburg, Germany) in PBS), and dehydrated through 25%, 50%, 75%, and 100% methanol (Merck, Darmstadt, Germany) in PBT for 3 min each at room temperature. The embryos were stored in 100% methanol at −20°C or rehydrated through a reversed methanol in PBT series for microscopy. The embryos were mounted in 3% methylcellulose (Roth, Karlsruhe, Germany) and photographed with an Axioplan stereomicroscope (Zeiss, Jena, Germany) using AxioVision software (Zeiss, Jena, Germany). The criterion for the classification of ‘mild’ or ‘severe’ phenotypes was the degree of altered somitogenesis (kinked tails versus truncated or no tails, respectively) and the presence and size of pericardial edema (present and small versus present and large, respectively). Embryos with small pericardial edema resembled the mild category of cortisol injected zebrafish embryos [15], while the embryos classified here as ‘severe’ showed pericardial edema like those of the moderate and severe category after cortisol microinjection [15]. Some morphological alterations like a deformed yolk sac and deformed or missing yolk sac extension were present in both categories.

### Steroid Extraction from Adult Zebrafish Holding Water and LC-MS/MS Analysis

Six adult zebrafish between 1.5 and 2 years old were placed for 16 hr in 800 mL freshly prepared fish water (75 mg/L NaHCO_3_, 18 mg/L sea salt, and 8.4 mg/L CaSO_4_ in H_2_O). Either mixed-gender groups composed of three females and three males or all-female and all-male groups were used. The experiment was repeated three times using the same group of fish with a resting period of at least 14 days in between. Conjugated reference steroids, cortisol-21-glucuronide (100 nM; Steraloids, Newport, Rhode Island, USA) and cortisone-21-sulfate (10 nM, Steraloids, Newport, Rhode Island, USA), were added to freshly prepared fish water and extracted following the same protocol. The extraction method and differential elution of the free and conjugated steroids were adapted from Payne *et al.* [39]. Solid phase extraction (SPE) cartridges (Strata C18-E, 200 mg/6 mL, Phenomenex, Aschaffenburg, Germany) were equilibrated twice with 2 mL methanol and twice with 2 mL water. Afterwards, 750 mL fish holding water was extracted using a vacuum manifold. To separate the conjugated from the unconjugated steroids, a differential elution was performed by first eluting the conjugated steroids with 300 µL 47% methanol in water (v/v) three times. Subsequently, the free steroids were eluted with 300 µL methanol three times. Both sets of eluates were evaporated to dryness. The fraction containing the free steroids was reconstituted in 300 µL methanol and stored at −20°C until analysis by LC-MS/MS. The fraction containing the conjugated steroids was reconstituted in 500 µL 75 mM NaP_i_ buffer pH 6.8. Forty-one units (U) β-glucuronidase (purified from *E. coli*, Sigma-Aldrich, Hamburg, Germany) was added, and the mixture was incubated overnight at 37°C with shaking. After the digestion of the steroid-glucuronide conjugates, steroids were extracted again by SPE. Strata C18-E 100 mg/1 mL cartridges (Phenomenex, Aschaffenburg, Germany) were equilibrated twice with 1 mL methanol and twice with 1 mL water, and the sample was applied. After washing with 500 µL water, the conjugated steroids were eluted twice with 200 µL 47% methanol before the free steroids (the formerly glucuronidated steroids) were eluted with 200 µL of methanol twice. The fractions were evaporated to dryness, and the free steroids were reconstituted in 300 µL methanol and stored at −20°C until analysis. The conjugated steroids were reconstituted in 500 µL 75 mM NaP_i_ buffer pH 5.1, and 7 U sulfatase (crude extract) of *Helix pomatia* (Sigma-Aldrich, Hamburg, Germany) was added. The mixture was incubated with shaking for 4–5 h at 37°C. Subsequently, the steroids were extracted by SPE again as described in the previous step, omitting the elution step with 47% methanol. The eluate was evaporated to dryness, and the steroids reconstituted in 300 µL methanol and stored at −20°C until analysis.

The steroids were separated by HPLC on a reversed phase Synergi Fusion RP18 column (150×3 mm, 4 µm, Phenomenex, Aschaffenburg, Germany) in 28% acetonitrile (v/v) and 0.1% formic acid (v/v) in water as the running solvent at a flow rate of 0.45 mL/min. The steroids were detected by MS/MS using an API4000 QTrap instrument (Applied Biosystems, Carlsbad, California, USA) with the APCI ion source set to scan in multiple reaction monitoring mode controlled by Analyst 1.5.1 software. The source parameters were set as follows: Curtain Gas 20.0 psi, Collision Gas 5 psi, Nebulizer Current 3.0 psi, Temperature 600°C, Ion Source Gas 1 40.0 psi, Entrance Potential 10.0 V, and Collision Energy 33.0 V. Detected mass transitions (masses given in Da) were as follows: cortisol 363.2/121.1, cortisone 361.2/163.2, and 20α−/20β-hydroxycortisone 363.2/163.2 (isomers distinguished by retention time). The chromatograms obtained were evaluated with regard to the peak height of each analyte. Frequent injections of reference substances were used to verify a constant sensitivity throughout the sequential sample analysis.

### RNA Isolation and Reverse Transcription Quantitative Real-time PCR

For RNA isolation, collected zebrafish embryos were homogenized in 500 to 750 µL TRIzol Reagent (Invitrogen, Darmstadt, Germany) using a syringe and needle (20 G). The volume used depended on the age and number of embryos. To the samples in TRIzol, 2/10 volumes chloroform (Merck, Darmstadt, Germany) was added, and the samples were vigorously shaken for 15 sec and incubated at room temperature for 3 min. To separate the phases, the samples were centrifuged at 21,000×g at 4°C for 15 min. The aqueous phase was collected and mixed with 0.53 volumes 100% ethanol (Merck, Darmstadt, Germany). This solution was applied to a column of the RNeasy Mini Kit (Qiagen, Hilden, Germany) and further purified including DNase I digestion according to manufacturer’s instructions. The RNA concentration and purity were determined by spectrophotometry. Only samples with an OD_260 nm_/OD_280 nm_ >1.95 were used in subsequent PCR experiments.

One microgram RNA was used in cDNA synthesis according to the manufacturer’s protocol (RevertAid First Strand cDNA Synthesis Kit, Fermentas, St. Leon-Rot, Germany). Instead of the supplied Oligo-dT_18_ primer, an anchored oligo-dT_18_ primer (5′-TTTTTTTTTTTTTTTTTTVN-3′) was used at a final concentration of 0.5 µM to prime the cDNA synthesis.

To verify the morpholino-induced mis-splicing, reverse transcription PCR (RT-PCR) reactions were performed as described [36] using primers with binding sites in the first and fourth exons of the *hsd20b2* transcript (forward 5′-AGACAATGCAGAGTGCTGCTGG-3′ and reverse 5′-GCCCTCTGTGAAGTCTGCCTG-3′).

Primers for quantitative real-time PCR spanning at least one exon-intron boundary were designed using Primer3 software (http://biotools.umassmed.edu/bioapps/primer3_www.cgi) [40]. However, this approach could not be applied to the genes for the melanocortin 2 receptor (*mc2r*) and corticotropin-releasing factor (*crh*), which consist of only one and two exons, respectively. The identity of all amplicons was verified by Sanger sequencing using standard protocols. Database accession numbers, amplicon lengths, and primer sequences for all of the reference and target genes are listed in Table 1.

**Table 1:** Primer sequences for quantitative real-time PCR.

gene name	accession number (Ensembl)		primer sequences (5′ 3′)	amplicon length (bp)
*actb1*	ENSDART00000055194	for	AAGGCCAACAGGGAAAAGAT	110
		rev	GTGGTACGACCGGAGGCATAC	
*eef1a1l1*	ENSDART00000023156	for	CAAGGAAGTCAGCGCATACA	189
		rev	GCATCAAGGGCATCAAGAAG	
*rpl8*	ENSDART00000140039	for	CCCCTTTCGCTTCCTCTTT	185
		rev	GTCCTTCACGATTCCCTTGA	
*hsd20b2*	ENSDART00000100769	for	AATGGTTGAAAGGGGGAAAG	201
		rev	TTATGGGTCATGTTCGTGGA	
*hsd11b2*	ENSDART00000141211	for	GGGGGTCAAAGTTTCCACTA	165
		rev	TGGAAGAGCTCCTTGGTCTC	
*cyp11c1*	ENSDART00000061572	for	ATGAAGTGGCGCAGGATTT	215
		rev	CTCCACAGCCGAAATGAAG	
*star*	ENSDART00000016225	for	AACAAGGGCAAGAAGCTCTG	207
		rev	CCCCCATTTGTTCCATGTTA	
*crh*	ENSDART00000038290	for	TCTGTTGGAGGGGAAAGTTG	198
		rev	ATTTTGCGGTTGCTGTGAG	
*mc2r*	ENSDART00000077231	for	CTCCGTTCTCCCTTCATCTG	126
		rev	ATTGCCGGATCAATAACAGC	

Real-time PCR amplifications were performed in triplicate using Power SYBR Green PCR Mastermix (Applied Biosystems, Carlsbad, California, USA). Assays consisted of 10 µL Mastermix, 6 µL water, 1 µL of each primer at a final concentration of 0.5 µM and 2 µL cDNA diluted 1∶20 in water. The reactions were performed in 384-well plates (ThermoScientific, Waltham, Massachusetts, USA) on a TaqMan 7900HT cycler equipped with SDS2.3 software (Applied Biosystems, Carlsbad, California, USA). The following amplification protocol was employed: denaturation at 95°C for 10 min, amplification and quantification repeated 39 times at 95°C for 15 sec and 60°C for 1 min, and a melting curve program (95°C for 15 sec, 60–95°C with a heating rate of 0.1°C/sec and continuous fluorescence measurement). The cycle threshold (C_T_) value was determined by SDS2.3 software as the cycle at which the fluorescence rose markedly above the background fluorescence.

In cortisol challenge experiments, the fold changes of 20β-HSD type 2 (*hsd20b2)* expression were calculated according to Pfaffl [41] with normalization to one reference gene each. The β-actin reference (*actb1*) was suitable. In the physical stress assay, the normalization was performed using the BestKeeper software tool developed by Pfaffl *et* *al*. [42] to calculate the fold changes of the target genes. In this case, β-actin, elongation factor-1 α (*eef1a1l1*) and ribosomal protein l8 (*rpl8*) were used as the reference genes. With both normalization methods, the data was expressed as fold changes from untreated control samples.

### Enzyme Assay in Fish Embryos

Enzymatic assays for determination of the activity of 20β-HSD type 2 in morpholino-injected embryos were performed as described earlier [36]. Because the sample material was limited, only 10 embryos were used per reaction and the incubation time was prolonged to 24 hr. Briefly, the embryos were homogenized in 450 µL reaction buffer (100 mM NaP_i_ pH 7.3, 1 mM EDTA, 0.05% BSA) using syringe and needle (20G). To 450 µL embryo homogenate, 20 nM tritiated cortisone (American Radiolabeled Chemicals, Saint Louis, Missouri, USA) and 50 µL NADPH+H^+^ (5 mg/mL in reaction buffer, Fluka, Buchs, Switzerland) were added and the samples were incubated at 28°C for 24 hr. Assays were performed in four replicates. Reactions were terminated by addition of 100 µL stop solution (0.5 M ascorbic acid, 1% acetic acid (v/v) in methanol) and the steroids extracted using solid phase extraction cartridges as described [36]. Steroids were analyzed by HPLC using an Allure Biphenyl column (3 µm, 50×2.1 mm, Restek, Bad Homburg, Germany) with 23% acetonitrile in water (v/v) as mobile phase. Conversion rates were obtained after integration of chromatograms as described [36].

### Reporter Gene Experiments

The coding sequence of the zebrafish MR (NM_001100403) was synthesized by Genscript (Piscataway, New York, USA) and subcloned into the vector pCS2+ by GeneArt (Life Technologies, Darmstadt, Germany) to obtain the expression plasmid zfMR_pCS2+. The expression plasmid for the zebrafish GRα (zGRα_pCS2+) and the reporter plasmid pMMTV-*luc* were kindly provided by Dr. Marcel Schaaf (Institute of Biology, Leiden, Netherlands). For reporter gene experiments, COS-1 cells (ATCC, Wesel, Germany) were maintained in DMEM medium (PAA, Pasching, Austria) supplemented with 10% fetal bovine serum (PAA, Pasching, Austria), 100 U/mL penicillin, and 100 µg/mL streptomycin (Invitrogen, Darmstadt, Germany) at 37°C and 5% CO_2_ in a humidified atmosphere. The cells were seeded at 5×10^4^ cells per well in 12-well plates and incubated for 24 hr. Cells were transfected with the expression plasmids using X-tremeGENE HP transfection reagent (Roche, Mannheim, Germany) according to the manufacturer’s protocol. Plasmids encoding the receptors were co-transfected with the reporter plasmid pMMTV-*luc* and the plasmid pGL4.74[*hRluc*/TK] (for normalization; Promega, Mannheim, Germany) at the ratio pGL4.74 : pCS2+ : pMMTV-*luc* 1∶ 10∶ 50. Cells were incubated for an additional 24 hr, the growth medium was replaced, and the steroids (cortisol, cortisone, aldosterone [all from Sigma-Aldrich, Hamburg, Germany], and 20β-hydroxycortisone [Steraloids, Newport, Rhode Island, USA]) dissolved in methanol were added at final concentrations of 0.1, 1, 10, 100, and 1000 nM. After the cells had been incubated an additional 24 hr, the cells were lysed, and the firefly and renilla luciferase activity were determined with the Dual-Luciferase Reporter Assay System (Promega, Mannheim, Germany). Bioluminescence was measured with the GloMax Multi Detection System (Promega, Mannheim, Germany) after the injection of each luciferase assay reagent. Firefly luciferase signals were normalized to renilla luciferase, and the background signal of methanol-treated cells was subtracted. The three technical replicates were averaged, and the mean values were calculated from three biological replicates. The data were plotted and fit according to a simple ligand-binding model by SigmaPlot 12.0 (Systat Software, Erkrath, Germany).

### Statistical Analyses

For all qPCR experiments, statistical significance was tested by using the REST2009 software [43], which is based on randomization and hypothesis tests. We increased the default value of 2000 iterations to 5000 iterations to achieve better-quality data. The results of the 20β-HSD type 2 activity in morpholino knock down animals were evaluated with Student’s t-test using SigmaPlot 12.0 (Systat Software, Erkrath, Germany). The appearance of the developmental phenotype in cortisol challenged morphants was tested for statistical significance by Fisher’s Exact test for count data. This test was conducted using the R software package [44].

## Results

### Cortisol Treatment Led to an Increase in *hsd20b2* and *hsd11b2* mRNA

**Figure 2:**
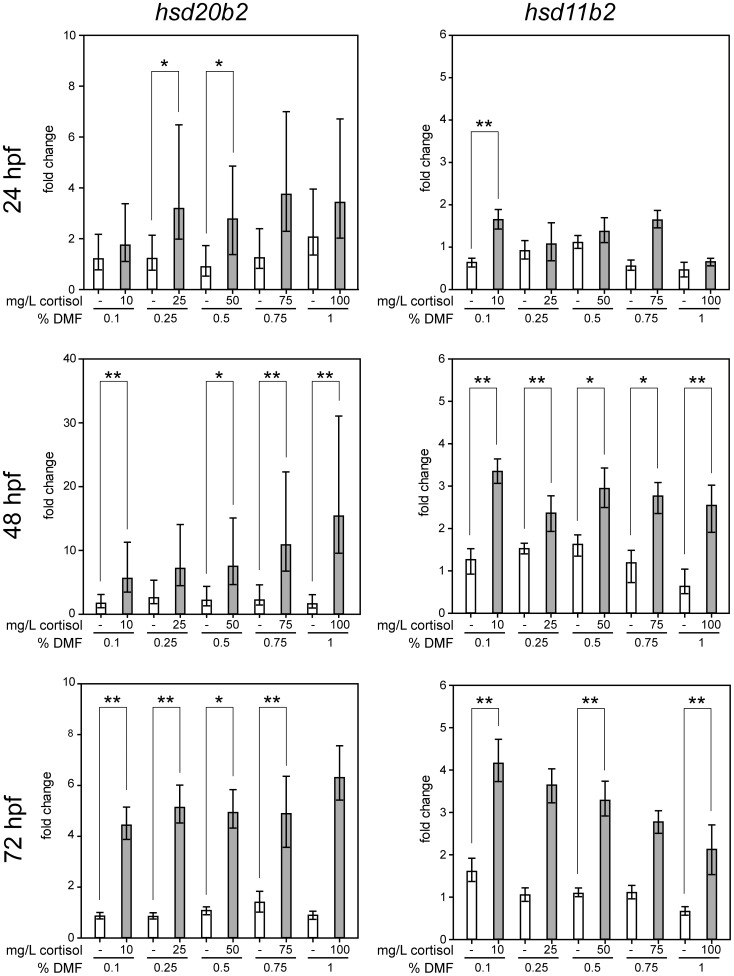
mRNA expression of *hsd20b2* and *hsd11b2* is increased by cortisol treatment. The effects of cortisol and its vehicle DMF were examined in embryos at 24 hpf and 48 hpf and in larvae at 72 hpf. Final cortisol concentrations of 10, 25, 50, 75, and 100 mg/L are equivalent to 27.5, 69.0, 137.9, 206.9, and 275.9 µM, respectively. The fold changes of three replicates were calculated after normalization to β-actin and compared with untreated fish embryos. White bars-the gene of interest in DMF treated fish; grey bars-the gene of interest in cortisol treated fish. Mean values with standard deviations are shown. Significance levels are indicated as follows: * p<0.05, ** p<0.01.

Zebrafish 20β-HSD type 2 was identified and characterized by us in a previous study [36]. We suggested a role for this novel enzyme in glucocorticoid catabolism and potentially in stress response. To determine if 20β-HSD type 2 expression is influenced by cortisol, we challenged zebrafish embryos with cortisol at varying concentrations in the range of 27.5 to 275.9 µM. As we attempted to elucidate the mechanism of 20β-HSD type 2 action, we challenged the zebrafish embryos with cortisol concentrations that were significantly higher than physiological levels. Using quantitative real-time PCR analyses, we observed an up-regulation of *hsd20b2* expression (Figure 2). In 24 hpf embryos, a 2- to 7-fold induction was detected, while in 48 hpf embryos, *hsd20b2* was significantly induced up to 18-fold after treatment with 100 mg/L (275.9 µM) cortisol. The level remained elevated 4- to 6-fold in 72 hpf old zebrafish larvae and at this stage the expression of *hsd20b2* was significantly higher than in DMF treated fish at nearly all cortisol concentrations applied. In the cortisol concentration range used, we observed no dosage dependency of the *hsd20b2* induction. Quantification of 11β-HSD type 2 mRNA showed that this gene is also up-regulated at all stages, though only in the moderate range of a 2- to 4-fold induction. We observed significant changes in expression of *hsd11b2* mostly starting at 48 hpf. *Hsd11b2* was also not induced in a dose-dependent manner; in 72 hpf larvae alone, the degree of up-regulation was negatively correlated with the cortisol concentration.

**Figure 3:**
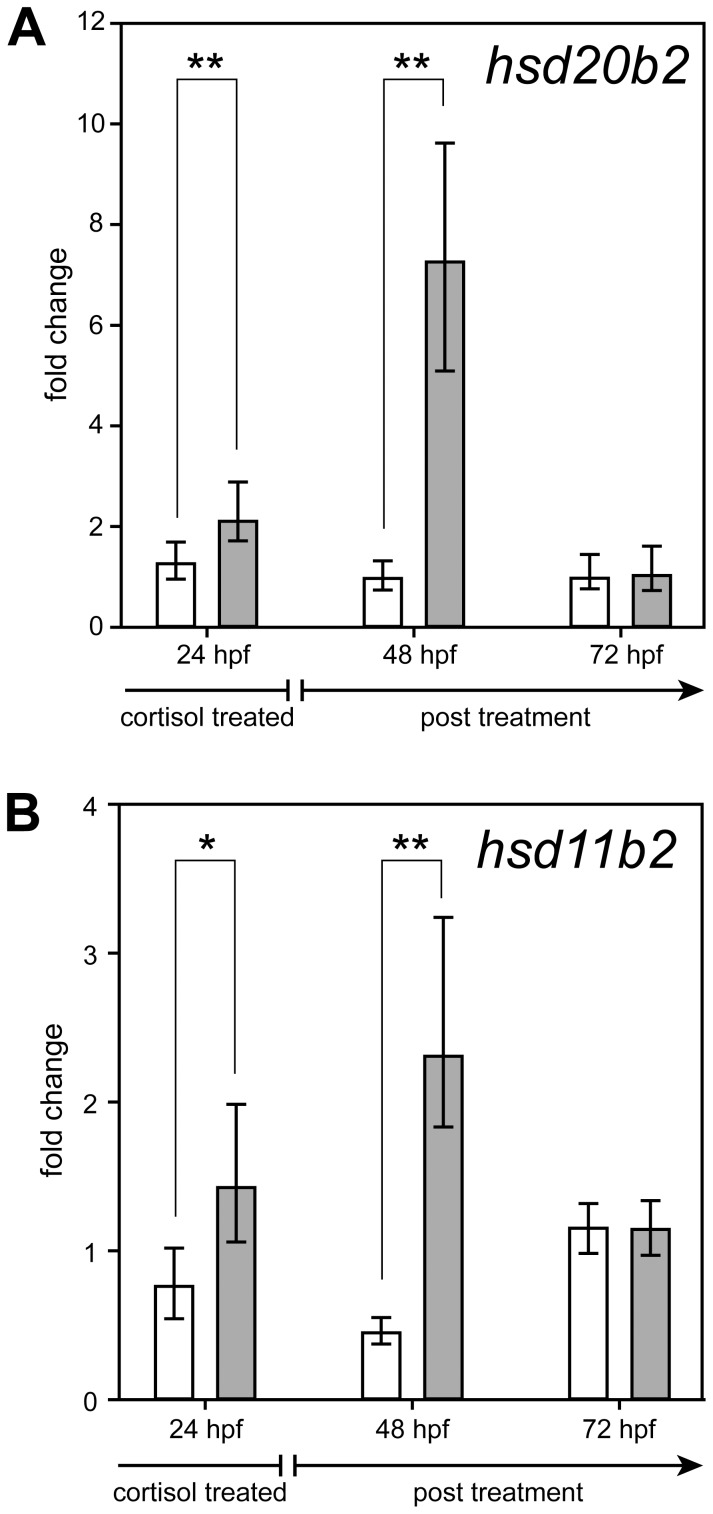
3–24 hpf exposure to cortisol up-regulates *hsd20b2* and *hsd11b2* mRNA in 24 and 48 hpf larvae. Zebrafish embryos were treated with cortisol and its vehicle DMF until 24 hpf and were then placed in cortisol-free embryo water. The fold changes of *hsd20b2* (A) and *hsd11b2* (B) expression in three replicates were calculated after normalization to β-actin and compared with untreated fish embryos. Mean values with standard deviations are shown. White bars-0.1% DMF; grey bars-50 mg/L cortisol (137.9 µM). Significance levels are indicated as follows: * p<0.05, ** p<0.01.

Inspired by these results, we determined whether a shorter cortisol treatment would suffice to induce 20β-HSD type 2 expression, and whether up-regulation induced this way would persist after cortisol removal. For the 3–24 hpf exposure, zebrafish embryos were treated with 50 mg/L cortisol (137.9 µM) until 24 hpf and were kept afterwards in untreated egg water until 72 hpf. Quantification of *hsd20b2* and *hsd11b2* mRNA demonstrated a significant increase of both transcripts (approximately 2-fold) in cortisol-treated embryos at 24 hpf. Twenty-four hours post treatment in the 48 hpf embryos, the induction of both genes was even higher (8-fold for *hsd20b2* and 2.5-fold for *hsd11b2*) but had decreased to normal levels at 72 hpf 48 hours after the cortisol challenge (Figure 3). Both *hsd20b2* and *hsd11b2* displayed a robust induction of expression upon both 3–72 hpf and 3–24 hpf cortisol treatment.

### Physical Stress Increased *hsd20b2* Expression

**Figure 4:**
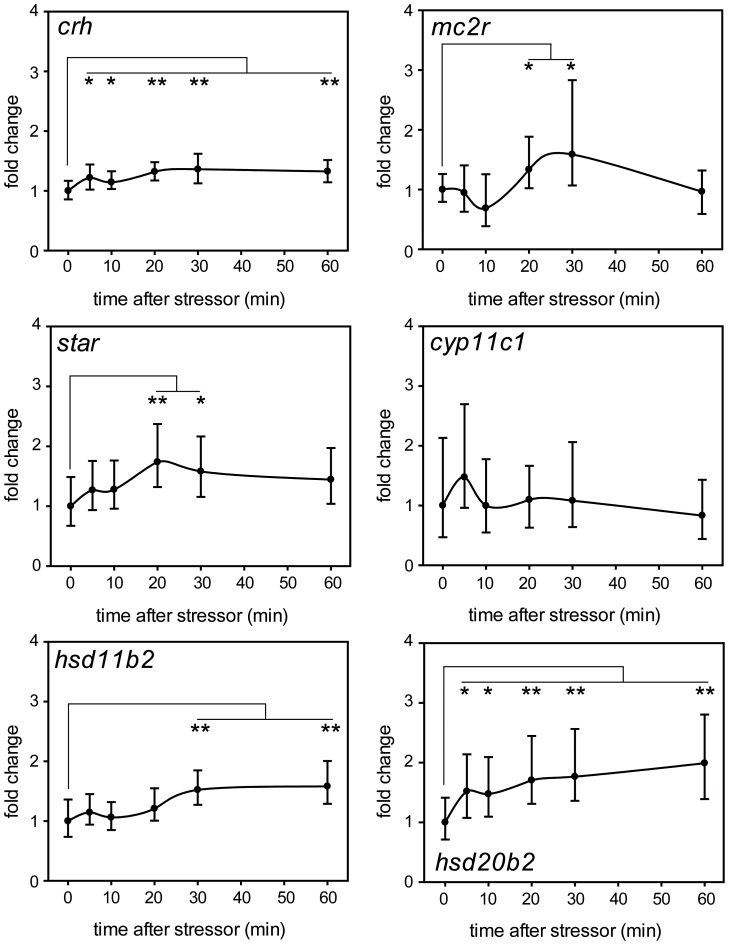
Challenge of 5 dpf zebrafish larvae with a physical stressor up-regulates *hsd20b2* and *hsd11b2* mRNA. The fold changes of target genes from six biological replicates were calculated after normalization to the geometric mean of three reference genes and compared with unstressed fish. The time courses for *crh*, *mc2r*, *star, cyp11c1, hsd11b2,* and *hsd20b2* are shown as mean values with standard deviations and significant changes to unstressed fish are indicated as follows: * p<0.05, ** p<0.01.

To determine if *hsd20b2* is up-regulated upon a stressor regimen independent of externally applied cortisol, we challenged 5 dpf zebrafish larvae with a physical stress composed of swirling the larvae for 30 sec. Samples were collected at different time points after the stress to analyze the time-dependent changes in expression levels. In this experiment, genes at different positions in the hypothalamus-pituitary-interrenal axis (HPI axis) were quantified to ensure accurate stress perception and transmission. The mRNA level of the first transmitter CRH in the hypothalamus increased slightly but significantly after the swirling stress and remained at this level for 60 min (Figure 4). The expression level of the melanocortin 2 receptor *mc2r* fluctuated during the first 10 min after the stressor, increased significantly during the next 20 min and subsequently decreased to the mRNA level of unstressed fish. *Mc2r* displayed a high variability between different samples. The transcript amount of steroid acute regulatory protein (*star*) increased 1.7-fold during the first 20 min after the stress and remained at this level until 60 min post-challenge. The expression levels of the gene encoding 11β-hydroxylase (*cyp11c1*) exhibited the highest inter-individual variability after the stressor. Some samples showed strong up-regulation of *cyp11c1* shortly after the stressor, while others displayed a marked decrease. Averaging these observations resulted in no significant change to the mean levels throughout the experiment (Figure 4). The mRNA of 11β-HSD type 2, the enzyme that catalyzes the inactivation of cortisol, steadily increased 1.5-fold 60 min post challenge compared with the levels in the unstressed fish. *Hsd20b2* was already induced 1.6-fold 5 min after the stressor, and the mRNA levels increased to 2.2-fold 60 min after the stressor (Figure 4). Neither the *hsd20b2* nor the *hsd11b2* transcript decreased to unstressed levels throughout the experiment. Although not as pronounced as the up-regulation after the artificial cortisol treatment, the expression of *hsd20b2* and *hsd11b2* was significantly induced after the application of a stressor.

### Cortisol-challenged *hsd20b2* Morphants Displayed a Developmental Phenotype

A transient knock down of *hsd20b2* in zebrafish embryos was performed to determine if 20β-HSD type 2 is essential to glucocorticoid removal and the survival of the developing embryo. Therefore, a morpholino was designed to target the donor splice site of exon 2. Morpholino-induced mis-splicing should result in an *hsd20b2* transcript that lacks exon 2, which would result in the translated 20β-HSD type 2 lacking the TGx_3_GxG motif of the cofactor binding site. The loss of this motif is supposed to render the enzyme totally inactive. Morpholinos were first injected at varying concentrations to ensure a sufficient knock down, which was verified by assaying mRNA levels and enzymatic activity. By RT-PCR, wild-type *hsd20b2* mRNA yielded a PCR fragment of 343 bp, while the mis-spliced PCR product was only 269 bp. Figure 1 B shows that upon injection of 375 µM morpholino, the wild-type PCR product decreased, while the shorter mis-spliced product increased. The amount of mis-spliced product could be increased by injecting 500 µM morpholino. However, monitoring the knock down efficiency at the enzymatic level (Figure 1 C) showed that injection of 500 µM morpholino did not further reduce 20β-HSD type 2 activity. Therefore, we chose to inject 375 µM instead of 500 µM morpholino in the subsequent experiments to reduce the possibility of toxic effects on zebrafish development.

**Figure 5:**
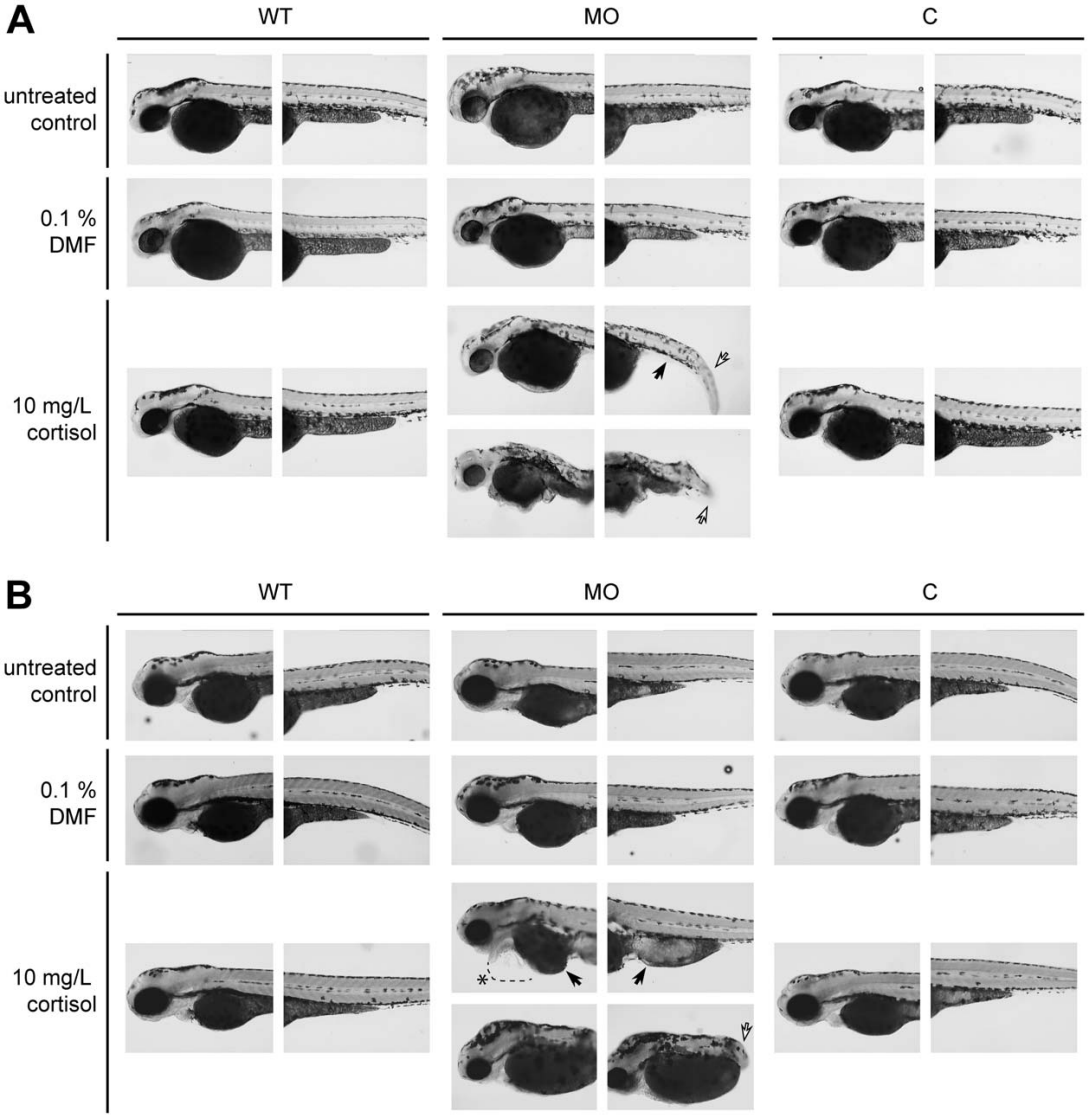
20β-HSD type 2 morphants display developmental abnormalities upon cortisol treatment. Animals are presented from a lateral view, with the anterior end at left. Representative specimens of the ‘mild’ and the ‘severe’ phenotype of the cortisol-treated 20β-HSD type 2 morphants are shown. (A) Embryos at 48 hpf and (B) at 72 hpf. Black arrows point to yolk deformations, white arrows point to altered somitogenesis resulting in kinked or truncated tails, and the asterisk denotes pericardial edema. WT-wild-type; MO-morpholino; C-control morpholino. Magnification 5x.

Despite the knock down of 20β-HSD type 2, the morphants exhibited no developmental abnormalities under normal culture conditions. To test our hypothesis that 20β-HSD type 2 is involved in cortisol catabolism and stress response, we investigated the effect of externally applied cortisol to the morphants, thereby mimicking stress. Because a cortisol treatment with 10 mg/L up-regulated *hsd20b2* mRNA in wild-type zebrafish embryos without inducing a visible developmental phenotype, this concentration was chosen to challenge the 20β-HSD type 2 morphants. Abnormalities manifested at 48 hpf and were characterized by an altered size and shape of both the yolk-sac and yolk-sac extension, altered somitogenesis, and kinked or shortened tails (Figure 5). The phenotypes could be categorized as a ‘mild’ or a ‘severe’ form, where the severe phenotype displayed strongly truncated tails. The described developmental abnormalities persisted until 72 hpf, but at this stage, strong pericardial edema was also observed (Figure 5, Table 2).

**Table 2:** Distribution of phenotypes in *hsd20b2* morphant zebrafish larvae.

48 hpf-untreated controls						
	phenotype					
animals	none (%)	mild (%)	p-value (mild)	severe (%)	p-value (severe)	n
WT	100±0	0±0		0±0		135
MO	100±0	0±0	0.28	0±0	1	87
C	99±2	1±2		0±0		85
**48 hpf-0.1% DMF**						
	**phenotype**					
**animals**	**none (%)**	**mild (%)**	**p-value (mild)**	**severe (%)**	**p-value (severe)**	**n**
WT	100±0	0±0		0±0		179
MO	99±2	1±1	0.26	1±1	0.25	91
C	100±0	0±0		0±0		114
**48 hpf-10 mg/L cortisol**						
	**phenotype**					
**animals**	**none (%)**	**mild (%)**	**p-value (mild)**	**severe (%)**	**p-value (severe)**	**n**
WT	100±0	0±0		0±0		153
MO	52±21	33±19	<2.2 e-16	16±5	9.75 e-12	104
C	96±5	5±5		0±0		111
**72 hpf-untreated controls**						
	**phenotype**					
**animals**	**none (%)**	**mild (%)**	**p-value (mild)**	**severe (%)**	**p-value (severe)**	**n**
WT	100±0	0±0		0±0		146
MO	91±11	4±4	0.013	4±7	0.0083	83
C	99±2	1±2		0±0		77
**72 hpf-0.1% DMF**						
	**phenotype**					
**animals**	**none (%)**	**mild (%)**	**p-value (mild)**	**severe (%)**	**p-value (severe)**	**n**
WT	100±0	0±0		0±0		188
MO	94±7	5±7	0.002	1±1	0.24	88
C	95±7	5±7		0±0		102
**72 hpf-10 mg/L cortisol**						
	**phenotype**					
**animals**	**none (%)**	**mild (%)**	**p-value (mild)**	**severe (%)**	**p-value (severe)**	**n**
WT	98±3	2±3		0±0		160
MO	37±20	44±12	<2.2 e-16	19±7	<2.2 e-16	105
C	86±12	14±12		0±0		110

Abbreviations: hpf-hours post fertilization; MO-morpholino-injected fish; C-control morpholino-injected fish; n-number of larvae. The significant appearance of the phenotype as determined by Fisher’s Exact Test is indicated by a p-value below 0.001. Data were pooled from four experiments and expressed as means ± SD.

### 20β-hydroxycortisone Activates Neither Zebrafish GRα nor MR

**Figure 6:**
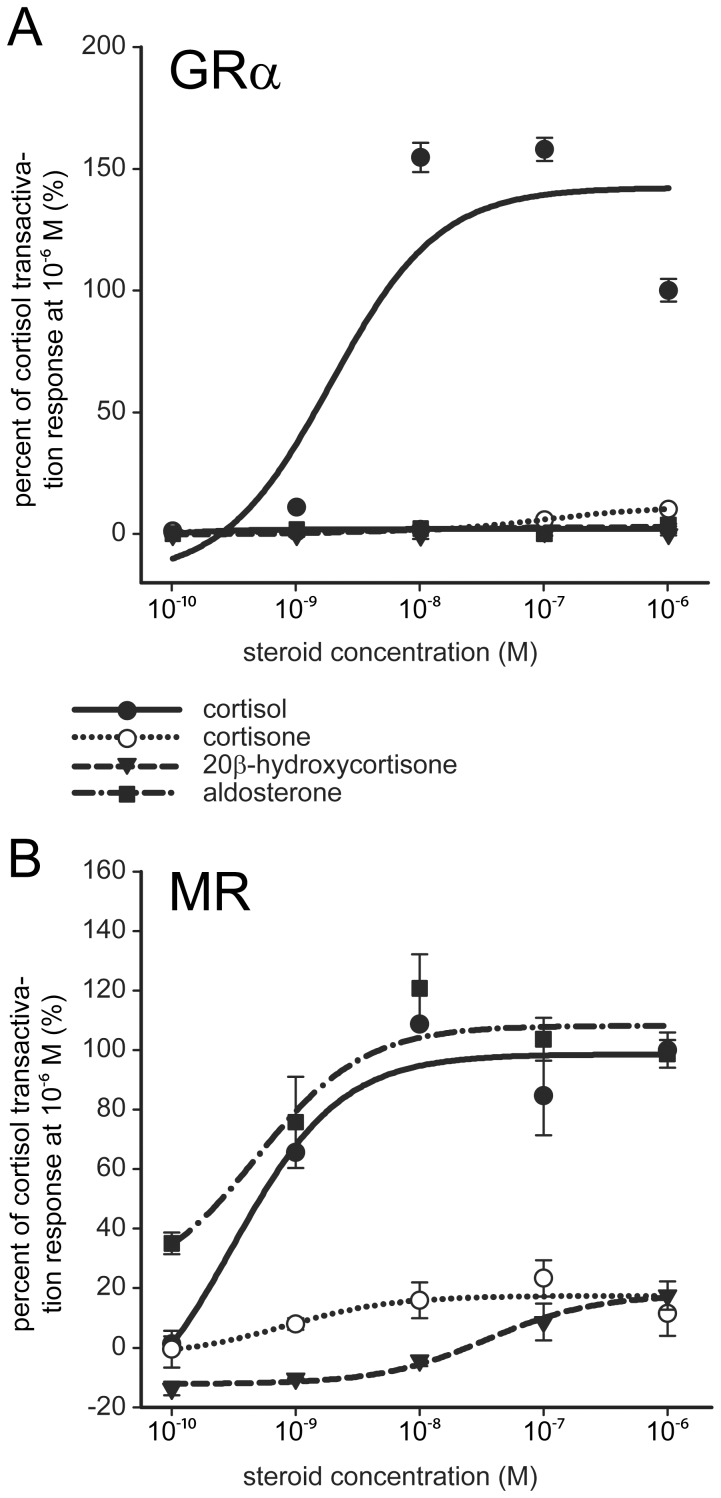
20β-hydroxycortisone is not a physiological ligand for zebrafish GRα or MR. An analysis of reporter gene activation in COS-1 cells mediated by zebrafish GRα (A) and zebrafish MR (B) using four different steroidal ligands. Firefly luciferase values were normalized to renilla luciferase and corrected for background. Mean values with standard deviations are shown.

To elucidate whether 20β-hydroxycortisone plays a role in steroid signaling by binding and activating glucocorticoid and mineralocorticoid receptors, we performed reporter gene experiments. Receptor expression vectors were co-transfected with a plasmid containing a luciferase gene driven by the MMTV promoter, which comprises several glucocorticoid response elements. COS-1 cells are frequently used for the reporter gene assays concerning GR because they lack an endogenous GR [23]. To ensure that the results obtained would be comparable, we used COS-1 cells for the MR experiments as well, even though this cell line expresses an endogenous MR. Hence, the results were corrected for the background caused by the endogenous receptor. For each receptor, dose-response curves were determined using four steroidal ligands, namely cortisol, cortisone, 20β-hydroxycortisone, and aldosterone (Figure 6). The zebrafish GRα was strongly activated by cortisol, while all the other steroids, including 20β-hydroxycortisone, induced no significant luciferase expression. The zebrafish MR was substantially activated by aldosterone and cortisol, whereas cortisone and 20β-hydroxycortisone stimulated it only slightly at concentrations far above physiological levels. The reporter gene experiments clearly demonstrated that 20β-hydroxycortisone does not bind or activate either the GRα or the MR at physiological concentrations.

### 20β-hydroxycortisone is a Major Excretion Product

**Figure 7:**
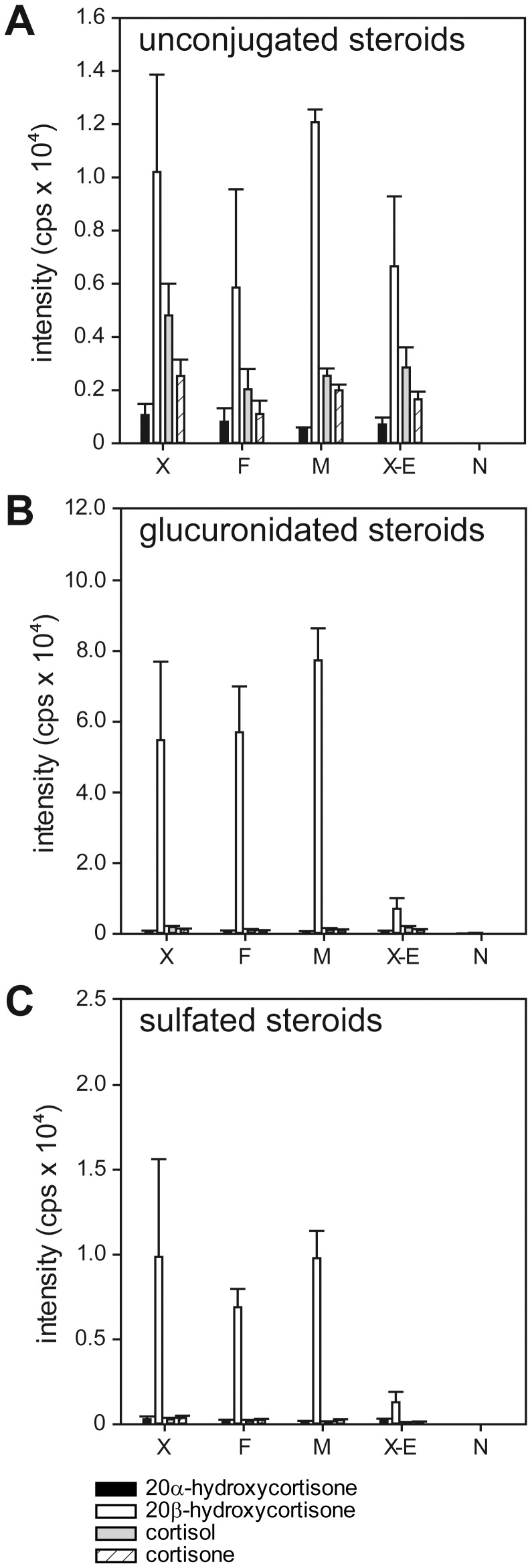
20β-hydroxycortisone is the most abundant glucocorticoid in extracts from adult zebrafish holding water. The measurement of four glucocorticoids in extracts from adult zebrafish holding water was performed by a separate LC-MS/MS analysis of each fraction: (A) unconjugated steroids; (B) glucuronidated steroids; (C) sulfated steroids. Each bar represents the mean value of three independent samples with standard deviations added. Sample abbreviations: X-mixed gender group; F-all female group; M-all male group; X-E-mixed gender group, without addition of digestive enzymes; N-water control without fish.

If 20β-HSD type 2 plays a role in cortisol catabolism, the reaction product 20β-hydroxycortisone might be labeled for excretion and released into the fish holding water. To determine whether 20β-hydroxycortisone is labeled with glucuronic acid and/or sulfates for excretion, we looked for glucocorticoids in the adult zebrafish holding water. After the extraction and fractionation of steroids from the water, the fractions of unconjugated, glucuronidated, and sulfated glucocorticoids were analyzed separately by LC-MS/MS. In our assay, conjugated glucocorticoids were only measurable after enzymatic hydrolysis. In all of the fractions, we detected four different glucocorticoids, with 20β-hydroxycortisone being the most abundant (Figure 7). In the unconjugated fraction, ample amounts of cortisol, cortisone, and 20α-hydroxycortisone were found aside from 20β-hydroxycortisone. In both the glucuronidated and sulfated steroid fractions, the amount of 20β-hydroxycortisone was an order of magnitude higher than all of the other glucocorticoids. We observed no significant sex-specific differences in the excretion pattern, although male fish seem to excrete slightly more 20β-hydroxycortisone than females do. Control extractions of glucocorticoids from fish holding water that had been twice digested with β-glucuronidase showed minor hydrolyzation of glucuronidated steroids after the first β-glucuronidase digestion (data not shown). Therefore, after one deglucuronization step, the actual amount of sulfated 20β-hydroxycortisone might be slightly lower than the value measured. Control fish water without fish demonstrated no steroid contamination. The results of the steroid extractions from the fish holding water subjected to the same extraction protocol but without the addition of β-glucuronidase or sulfatase exhibited only a minor carry-over of the free steroids. Both of the conjugated reference steroids, cortisol-21-glucuronide and cortisone-21-sulfate, were contaminated with free glucocorticoids but were only hydrolyzed by β-glucuronidase and sulfatase, respectively, demonstrating the functionality of the extraction method as well as the digestion protocol (data not shown). Altogether, these observations indicate that 20β-hydroxycortisone is indeed labeled with both glucuronic acid and sulfates and can be considered a major excreted glucocorticoid based on its high abundance in the conjugated steroid fractions.

## Discussion

Recently, we identified the novel enzyme 20β-HSD type 2 and hypothesized that the enzyme plays a role in cortisol catabolism in concert with 11β-HSD type 2 [36]. The results obtained substantiate our hypothesis that 11β-HSD type 2 and 20β-HSD type 2 are involved in cortisol catabolism and stress response. In this study, we present data concerning the cortisol induction of zebrafish 20β-HSD type 2 expression, the physiological implications of 20β-HSD type 2 knock down, and evidence that 20β-hydroxycortisone is an excreted glucocorticoid.

To analyze whether the regulation of 20β-HSD type 2 expression is cortisol-dependent, we treated zebrafish embryos with varying concentrations of cortisol. The chosen concentrations were higher than physiological cortisol levels due to three reasons: Firstly, we were primarily interested in elucidating the mechanistic role of 20β-HSD type 2 in zebrafish. To ensure the observation of a cortisol-dependent effect, we administered rather high concentrations. Secondly, comparable studies exposing zebrafish embryos to cortisol are rare. To date, there were only two publications [12], [17] exposing zebrafish embryos to cortisol and in these studies 100 mg/L cortisol were necessary to observe an effect. We designed our cortisol challenge experiments in accordance to these studies. Thirdly, the chorion of zebrafish embryos represents a major barrier for all kinds of chemicals. Studies on the permeability of steroids were so far not performed, but cryoprotective compounds like glycerol or DMSO do not diffuse easily across the chorion [45], [46]. Thus, to ensure that cortisol diffused into the embryo and exerted a measurable effect on the expression of 20β-HSD type 2 and 11β-HSD type 2, rather high concentrations of cortisol were chosen. Challenging wild-type embryos with cortisol up-regulated the known catabolic enzyme 11β-HSD type 2. Moreover, 20β-HSD type 2 was induced even more strongly. Neither gene’s up-regulation was dose-dependent, although in 72 hpf zebrafish larvae, 11β-HSD type 2 showed a negative correlation between the degree of up-regulation and the cortisol concentration. The reason for this observation is not clear but could be due to the rather high cortisol concentrations used in our experimental setup. A positive correlation of dosage and up-regulation of both genes might be detectable if cortisol was applied within a physiological concentration range.

Even a shorter cortisol exposure (3–24 hpf) of the zebrafish embryos significantly increased the levels of *hsd20b2* and *hsd11b2* expression. Twenty-four hours after the removal of the cortisol from the fish medium, the mRNA levels of both enzymes in 48 hpf embryos were even higher than the levels observed while cortisol remained in the fish medium. Despite the cortisol removal from the medium, the cortisol concentration in the fish embryo was likely still elevated, leading to the continued overexpression of both 11β-HSD type 2 and 20β-HSD type 2. The increased amount of both enzymes is likely necessary to catabolize excess cortisol to regain homeostasis. Alternatively, this effect may also reflect the long half life of both the *hsd20b2* and *hsd11b2* transcripts. In the 72 hpf larvae 48 hours post challenge, the mRNA of both enzymes decreased to wild-type levels. This observation might indicate that cortisol inactivation has proceeded and the cortisol concentration in the fish reached a level which is insufficient to stimulate the *hsd11b2* and *hsd20b2* expression.

To elucidate whether 11β-HSD type 2 and 20β-HSD type 2 are up-regulated by stress independently of artificial cortisol treatment, we challenged zebrafish larvae with a physical stressor. This stressor consisted of swirling and was adapted from Alsop and Vijayan [11], who have shown that the stress axis is fully functional by 97 hpf and that cortisol concentrations are significantly elevated as early as 5 min after the 30 sec swirling stress. We challenged 5 dpf zebrafish larvae with a 30 sec swirling stressor and analyzed the expression of the HPI axis genes at various time points after the stressor. Most HPI axis genes were up-regulated upon physical stress (e.g., *crh*, *mc2r*, and *star*), while the levels of *cyp11c1* followed no visible trend due to high inter-individual variability. *Mc2r* alone decreased to unstressed levels 60 min after the stress, whereas *crh* and *star* remained slightly elevated throughout the experiment. These observations demonstrate an activated HPI axis and successful stress transmission. However, the intensity and duration of up-regulation deviated slightly from the results obtained by subjecting adult zebrafish to a 60 min vortex stressor, after which *mc2r*, *star*, and *cyp11c1* exhibited the highest expression after 20 min, and *crh* had already peaked after 10 min [47]. The mRNA levels of all four genes decreased to unstressed levels by 60 min [47]. These discrepancies are most likely due to the different approaches used in the stress challenges and the different sources of RNA in our study compared with Fuzzen *et al.* (2010) [47]. We subjected 5 dpf zebrafish larvae to a short-term stressor, while Fuzzen *et al*. (2010) applied a long-term stressor to adult zebrafish. In addition, Fuzzen *et al*. (2010) quantified the HPI axis genes in the head kidney alone, which contrasts with our approach of quantifying the mRNA of the whole larvae. Nevertheless, the general results of both approaches are comparable, and any differences may well reflect regulatory processes that differ between larval and adult fish. Regarding the mRNA of the cortisol-inactivating enzyme *hsd11b2*, Fuzzen *et al.* (2010) detected a peak after 20 min and, after 60 min, observed mRNA levels similar to those under unstressed conditions. After subjecting the 5 dpf larvae to the short-term stressor, we observed a slight but steady increase of *hsd11b2* mRNA that reached 1.5-fold after 30 min and did not decrease until 60 min post-stress. The potentially catabolic enzyme, *hsd20b2*, had already increased 1.6-fold 5 min after the stressor, and it reached a 2.2-fold induction after 60 min. The mRNA of both enzymes did not decrease to unstressed levels during the experiment, indicating that cortisol inactivation and putative excretion were probably involved in the biochemical stress reduction even 60 min after the stressor was perceived. The prolonged up-regulation of both catabolic enzymes could protect the larvae from the adverse effect of stress on their development.

*Hsd20b2* expression analysis in early zebrafish development revealed that its mRNA was maternally supplied to the zygote [36]. To elucidate the impact of an *hsd20b2* knock down on zebrafish development, we designed a splicing morpholino. This morpholino exerted its action after the onset of zygotic transcription to avoid the potentially lethal effects of a translation-blocking morpholino, whose action would arise directly after its injection. The knock down of *hsd20b2* was verified by the significant decrease of both the 20β-HSD type 2 mRNA and activity levels in the morphant fish larvae. Under normal culturing conditions, the morphants displayed no developmental phenotype. However, challenging *hsd20b2* morphants with a cortisol concentration sufficient to induce *hsd20b2* expression but not to visibly harm wild-type fish embryos led to developmental changes. Phenotypical abnormalities appeared first at 48 hpf and persisted and, in some cases, intensified until 72 hpf. The observed developmental abnormalities of the challenged morphants resembled a ‘cortisol phenotype’ previously described by Hillegass *et al.* [17]. Comparable tail deformations have also been observed after morpholino-induced knock-down of the GRα [13], [14], and pericardial edema have formed upon microinjection of cortisol into zebrafish embryos to mimick the influence of maternal stress on the fish progeny [15]. Our results for the *hsd20b2* morphants hint at an impaired cortisol catabolism pathway assuming a vital role for 20β-HSD type 2 in reducing cortisol concentrations in concert with 11β-HSD type 2.

Steroids exert their effects on the gene expression mediated by members of the nuclear receptor family. Cortisol is the unique ligand for the GR in teleost fish [7], [23] and is also able to activate the MR [28], [29]. To rule out the involvement of 20β-hydroxycortisone in steroid signaling, reporter gene experiments were performed that took zebrafish GRα and MR into account. While zebrafish GRα has already been shown to bind cortisol with an EC_50_ value of 10 nM [23], the endogenous ligand for zebrafish MR is not yet known. Teleost fish lack the capability to synthesize aldosterone [27], but there is evidence that 11-deoxycorticosterone might be the ligand for MR in teleost fish [28], [29]. Our results for zebrafish GRα confirmed the high affinity binding of cortisol published earlier [23], additionally demonstrating that no other steroidal ligand tested here, including 20β-hydroxycortisone, was able to activate this receptor. As expected, the ligand spectrum of the zebrafish MR was broader than the spectrum of the GRα, as the MR was activated by aldosterone and cortisol. Cortisone and 20β-hydroxycortisone induced MR reporter gene expression to a negligible extent and only at high concentrations. This result showed plainly that physiological concentrations of 20β-hydroxycortisone do not bind or activate either GRα or MR; 20β-hydroxycortisone likely has no role in the steroid signaling pathways of either receptor.

Being that it is not involved in steroid hormone signaling, we investigated whether 20β-hydroxycortisone might be an excreted end product of cortisol catabolism. In humans, cortisol is primarily inactivated by 11β-HSD type 2 to yield cortisone [30], which is reduced at the C4–C5 double bond and at the C3 and C20 carbonyl groups. The products that result are subsequently either conjugated to increase their solubility in water or excreted in their free form via the urine. In fish, it is generally accepted that cortisol is metabolized along similar pathways [7]. Studies investigating glucocorticoid excretion in fish have been rare but indicate a more varied picture because fish can excrete steroids via the gills and the bile as well as the urine [33]–[35]. Steroid moieties in the bile of rainbow trout included tetrahydro-derivatives of cortisol and cortisone, 20β-cortolone, 5β-dihydrocortisone, cortisone, and cortisol as the major excretion products [33], [34]. Our analysis of glucocorticoids in zebrafish holding water revealed 20β-hydroxycortisone to be the most abundant steroid in both the conjugated and free steroid fractions. This high amount of 20β-hydroxycortisone in the conjugated fractions, whether glucuronidated and sulfated steroids, indicates that this steroid is predominantly labeled for excretion. This observation suggests a putative excretion pathway parallel to and independent of that described above. Instead of the reduction of the C4–C5 double bond followed by the reduction of C3 and C20 carbonyl groups, we found a shorter pathway for cortisol catabolism and excretion. This pathway involves only 11β-HSD type 2, 20β-HSD type 2, glucuronosyltransferase, and sulfatase and can be quickly activated upon a stressor. It can be speculated that both 11β-HSD type 2 and 20β-HSD type 2 are important in protecting the adult zebrafish as well as the developing zebrafish embryo from exogenous glucocorticoids, beside coping with endogenously produced cortisol upon stress.

In humans, 11β-HSD type 1 catalyzes the reactivation of cortisone to cortisol [48], [49]. 11β-HSD type 1 does not exist in zebrafish, but its ancestor, 11β-HSD type 3, was identified in the zebrafish genome and suggested to fulfill 11β-HSD type 1 functions [50], [51]. The action of 20β-HSD type 2 can be considered to reduce the amount of cortisone available for reconversion to cortisol by 11β-HSD type 3 [36]. However, up to now there is no experimental evidence published for the cortisone to cortisol conversion in zebrafish. Additionally, 11β-HSD type 3 failed to catalyze this reaction in our hands (data not shown). Thus, the question whether cortisone is indeed reactivated to cortisol remains unanswered. Furthermore, it can only be speculated that 20β-HSD type 2 is the enzyme responsible for catalyzing the efflux of cortisone out of the putative cortisol inactivation-reactivation cycle.

In summary, we have shown that zebrafish 11β-HSD type 2 and 20β-HSD type 2 transcripts are significantly up-regulated by stress signals in the form of either cortisol treatment or the application of an acute physical stressor independent of artificial cortisol application. Morpholino-induced knock down of *hsd20b2* caused no developmental phenotype under normal culturing conditions, but after challenging the morphants with cortisol, we observed abnormalities such as yolk deformations, altered somitogenesis, and pericardial edema previously described in connection with cortisol exposure [17] and disrupted cortisol signaling [13]–[15]. The cortisol-dependent regulation of 11β-HSD type 2 and 20β-HSD type 2 and the diminished capability of *hsd20b2* morphants to cope with an excess of cortisol demonstrates the concerted action of both enzymes in cortisol inactivation. Additionally, we have shown that the reaction product of 20β-HSD type 2, 20β-hydroxycortisone, does not bind to or activate either zebrafish GRα or MR. Instead of being involved in steroid signaling pathways, 20β-hydroxycortisone is excreted predominantly after its conjugation into the fish holding water. Therefore, 11β-HSD type 2, together with the novel 20β-HSD type 2, constitutes a rapid pathway in zebrafish to efficiently inactivate cortisol and excrete it after stressful situations.
